# Emotion recognition based on customized smart bracelet with built-in accelerometer

**DOI:** 10.7717/peerj.2258

**Published:** 2016-07-26

**Authors:** Zhan Zhang, Yufei Song, Liqing Cui, Xiaoqian Liu, Tingshao Zhu

**Affiliations:** 1 School of Computer and Control Engineering, University of Chinese Academy of Sciences, Beijing, China; 2 Institute of Psychology, Chinese Academy of Sciences, Beijing, China

**Keywords:** Emotion recognition, Wearable smart device, Smart bracelet, Accelerometer

## Abstract

**Background:** Recently, emotion recognition has become a hot topic in human-computer interaction. If computers could understand human emotions, they could interact better with their users. This paper proposes a novel method to recognize human emotions (neutral, happy, and angry) using a smart bracelet with built-in accelerometer.

**Methods:** In this study, a total of 123 participants were instructed to wear a customized smart bracelet with built-in accelerometer that can track and record their movements. Firstly, participants walked two minutes as normal, which served as walking behaviors in a neutral emotion condition. Participants then watched emotional film clips to elicit emotions (happy and angry). The time interval between watching two clips was more than four hours. After watching film clips, they walked for one minute, which served as walking behaviors in a happy or angry emotion condition. We collected raw data from the bracelet and extracted a few features from raw data. Based on these features, we built classification models for classifying three types of emotions (neutral, happy, and angry).

**Results and Discussion:** For two-category classification, the classification accuracy can reach 91.3% (neutral vs. angry), 88.5% (neutral vs. happy), and 88.5% (happy vs. angry), respectively; while, for the differentiation among three types of emotions (neutral, happy, and angry), the accuracy can reach 81.2%.

**Conclusions:** Using wearable devices, we found it is possible to recognize human emotions (neutral, happy, and angry) with fair accuracy. Results of this study may be useful to improve the performance of human-computer interaction.

## Introduction

The recognition of emotions plays an important role in human-computer interaction (Chen et al., 2015). If a computer could recognize emotions, it would be able to interact better with human being (Beale & Peter, 2008; Brave & Nass, 2003; Peter, Crane & Beale, 2006). In psychology, emotion is defined as a complex state that consists of a subjective experience (how we experience emotion), a physiological response (how our bodies react to emotion), and an expressive response (how we behave in response to emotion) (James, 1884; Smith & Lazarus, 1990). It suggests that the observable aspects of emotion (physiological and expressive components) could be used as indicators of emotional state, such as facial expressions, speech, physiological parameters, gestures, and body movements (Peter & Beale, 2008; Zhang et al., 2013).

Evidence exists that human emotions are expressed in walking (Karg, Kühnlenz & Buss, 2010; Montepare et al., 1999) to some extent. Michalak et al. (2009) found that both sadness and depression can be recognized through walking styles. Crane & Gross (2007) found that emotion is associated with body movements, including velocity, cadence, head orientation, and shoulder and elbow range of motion. Montepare et al. (1999) reported that human emotions can be identified through gait information. They found that among emotions (neutral, happy, sad, and angry), sad and angry can be identified more easily from gait information. Recently, a few studies have attempted to build computational models for human identification using gait (Han & Bhanu, 2006; Kale et al., 2004). However, computational methods of gait-based emotion detection have not yet been fully established. More importantly, human emotions change over time, only if we can access gait information ubiquitously, otherwise we are not able to implement real-time recognition of emotions. Traditional methods (e.g. self-report, interviewing, and observation) may lead to delayed reporting, which fail to meet the requirement.

The emergence of wearable smart devices shed light on this direction. Wearable smart devices are accessories incorporating computer and advanced electronic technologies, incorporated with various types of sensors. Taking the accelerometer sensor as an example, it can serve as a tool for tracking and recording a user’s daily movements. Lee & Mase (2002) found that it is possible to recognize human activities through the use of wearable sensors. Bao & Intille (2004) placed wearable biaxial accelerometers at different body positions to collect acceleration data for recognizing human activities. He & Jin (2008) recognized human activities through the use of triaxial accelerometers. Sun et al. (2010) and Kwapisz, Weiss & Moore (2011) recognized physical activities with the help of a smartphone with built-in sensors. These studies motivate us to collect acceleration data as a good representation of human activities, which may be beneficial to identify human emotions in real-time.

In this paper, we propose to recognize human emotions (neutral, happy, and angry) using wearable smart devices. We aim to build classification models for differentiating different emotions, using human gait data collected from a smart device with built-in accelerometer.

## Methods

The procedure of our work consists of four steps: (1) Data collection, (2) Data preprocessing, (3) Feature extraction and selection, and (4) Model training. Methods and procedures of this study have been approved by the Institutional Review Board of the Institute of Psychology, Chinese Academy of Sciences, H15010.

### Data collection

In this study, a total of 123 healthy postgraduate students (45 women and 78 men) were recruited from the University of Chinese Academy of Sciences (UCAS). All participants were required to wear a customized smart bracelet with built-in accelerometer that can track their movements. Specifically, the bracelet records movement behavior from 3-dimentional acceleration data (X axis: longitude; Y axis: latitude; Z axis: elevation).

**Figure 1:**
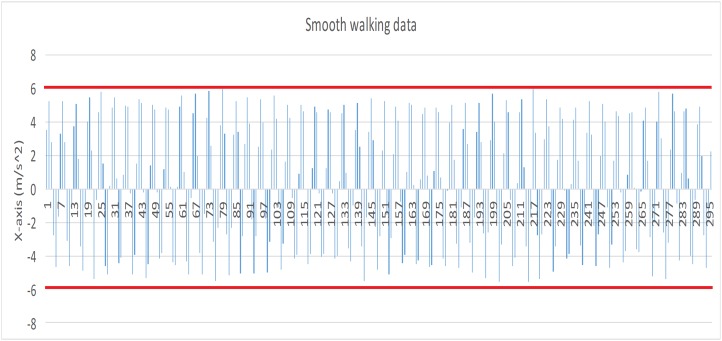
Data record produced by smooth walking (X axis).

**Figure 2:**
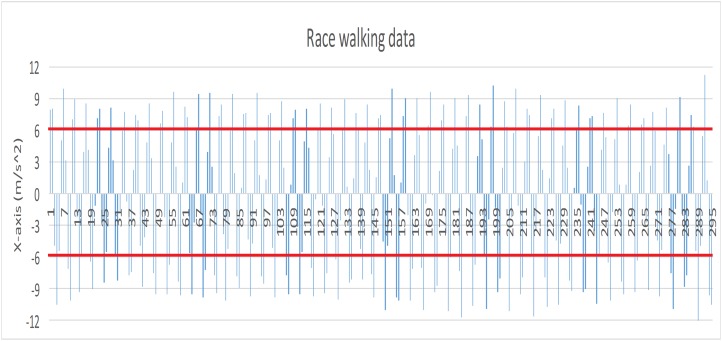
Data record produced by race walking (X axis).

Before the experiment, we tested the sensitivity of smart bracelet in different walking style. The absolute value of smooth walking data (X axis) (see Fig. 1 red lines) were less than 6 (m/s^2^), and many absolute value of race walking data (X axis) (see Fig. 2 red lines) were greater than 6 (m/s^2^). Results indicated that the bracelet is effective for differentiating smooth walking from race.

After validation, we run the experiment as follows. To avoid any environmental noise, we selected a quiet room to conduct the experiment. In the room, the floor was covered by a rectangular red carpet (length: 5 meters; width: 1 meter). All 123 participants were required to wear the smart bracelet on their right wrist and ankle, then walked back and forth on the carpet. In this study, the bracelet tracked a participant’s movements continuously (five times per second), which generated time series data for further analysis. Specifically, the experiment consists of three parts.

Firstly, participants were instructed to walk for two minutes, which served as walk in neutral emotion.

Secondly, all participants were required to rate their angry emotion by a 10-point Likert Scale (1 = Not angry to 10 = Extremely angry). They then watched a 2-min film clip to elicit angry (Yan et al., 2014). This clip tells a short story about a girl who was run over by a vehicle. As she lay bleeding on the road, at least two persons skirted around her boy and just ignored. Eventually, the girl died. After watching the clip, participants were required to walk for one minute, i.e., walk in angry as expected. After walking, they were asked to rate their emotion (angry) for the second time.

Thirdly, to minimize the effects of angry emotion, we conducted third part more than four hours after the second part. Participants were instructed to walk for two minutes, as neutral walking behaviors. They were required to rate their happy emotion by a 10-point Likert Scale (1 = A little happy to 10 = Extremely happy), and then watched a 1.5-min film clip to elicit the emotion of happy (Yan et al., 2014). This film clip is a funny cartoon. After watching the clip, participants were required to walk for another one minute, i.e., walk in happy, and they rated their happy emotion after then.

**Figure 3:**
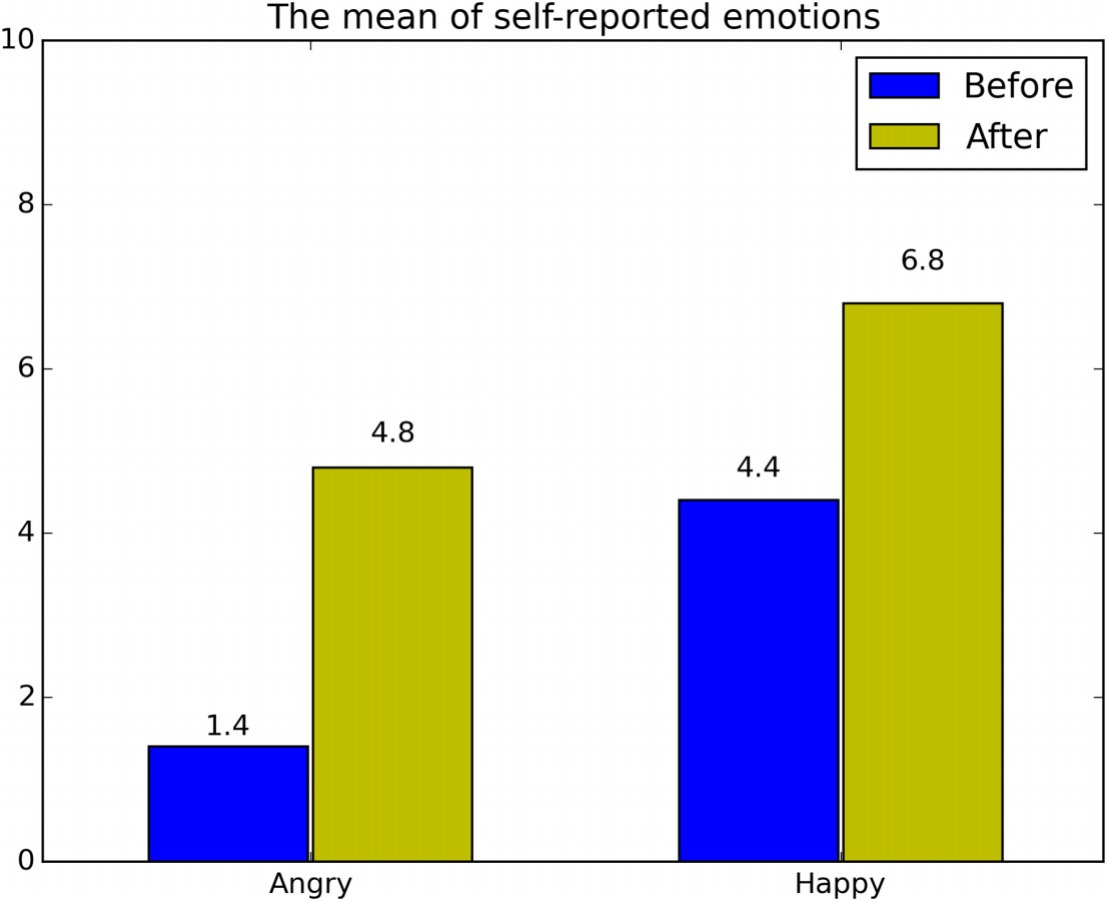
The mean of self-reported emotions (angry and happy).

The mean of self-reported angry and happy emotions was shown in Fig. 3. Results indicated that the angry and happy videos had evoked the desired emotional response.

To avoid cold-start effect of walk in neutral, we only examined a participant’s movements within the last minute. Therefore, for each one emotion condition, the time series has the same length (one minute).

### Data preprocessing

The data preprocessing is done as follows.

Firstly, as unexpected walking vibrations might cause noise in data collection, we used Moving Average Filter to address this issue.

Filters are functions that convert one time series into another. By choosing appropriate filter, certain patterns in the original time series can be eliminated in the new one. Moving Average Filter is a kind of low-pass filter, which removes high frequency components and yields an estimate of the slow-moving trend. It operates by averaging a number of points from the input signal to generate a new point in the output signal. The convert process is defined as:

(1) $$Output\;[i] = {1 \over w}\sum\limits_{j = 0}^{w - 1} I nput\;[i + j]$$

*Input* refers to the input signal (original time series data recorded by the bracelet), *Output* refers to the output signal (new time series data), and *w* refers to the number of points used to generate a new point. In this study, we set *w* ∈ {3, 5} (Cui, Li & Zhu, 2015; Ma, 2013).

Secondly, we reduced data redundancy. Since walking can be considered as a set of repetitive behaviors, a long-term data collection may lead to data redundancy, which can cause inefficiency in computation.

As the smart bracelet can track a participant’s movements continuously, we are able to get a time series composed of 300 (5 × 60 = 300) sets of 3-dimentional acceleration data (*x*, *y*, *z*) within one minute. To cope with such data effectively, we used sliding window (*windowsize* = 128) to divide the original time series into segments with equal length. Each segment can be regarded as a new time series (a data sample). In order to ensure an overlap ratio of 50% (Cui, Li & Zhu, 2015; Xue & Jin, 2011), the sliding step was set as 64. For example, for each user, the original time series {(*x*_1_, *y*_1_, *z*_1_), …, (*x*_300_, *y*_300_, *z*_300_)} can be divided into three segments, such as {(*x*_1_, *y*_1_, *z*_1_), …, (*x*_128_, *y*_128_, *z*_128_)}, {(*x*_65_, *y*_65_, *z*_65_), …, (*x*_192_, *y*_192_, *z*_192_)}, and {(*x*_129_, *y*_129_, *z*_129_), …, (*x*_256_, *y*_256_, *z*_256_)}. Each segment consists of 128 sets of 3-dimensional acceleration data. Because of the limited size (less than 128), the rest of data {(*x*_193_, *y*_193_, *z*_193_), …, (*x*_300_, *y*_300_, *z*_300_)} would be removed.

After preprocessing, we can reduce data redundancy and increase our sample size by three times, which is beneficial to build classification models.

### Feature extraction and selection

In this paper, three kinds of features are extracted: temporal domain, frequency domain and temporal-frequency domain features.

#### Temporal domain features

For any participant’s data on each one of three axes, we calculated skewness (*S^t^*), kurtosis (*K^t^*), and standard deviation (*σ*^*t*^), in which, *t* denotes the axis label (*t* ∈ {*x*, *y*, *z*}). In addition, we also estimated correlations between every two axes.

Specifically, skewness refers to the asymmetry of the probability distribution of data, which is defined as:

(2) $${S^t} = {{{1 \over n}\sum\limits_{i = 0}^{n - 1} {{{(x_i^t - {{\bar x}^t})}^3}} } \over {{{ \left( {1 \over n}\sum\limits_{i = 0}^{n - 1} {{{(x_i^t - {{\bar x}^t})}^2}} \right)}^{{3 \over 2}}}}}$$

where $x_i^t$ refers to acceleration data on the *t* axis, *n* refers to the width of a segment, and ${\bar x^t}$ refers to a mean value of all data on *t* axis.

Kurtosis measures the flatness of the probability distribution, which is defined as:

(3) $${K^t} = {{{1 \over n}\sum\limits_{i = 0}^{n - 1} {{{(x_i^t - {{\bar x}^t})}^4}} } \over {{{\left( {{1 \over n}\sum\limits_{i = 0}^{n - 1} {{{(x_i^t - {{\bar x}^t})}^2}} } \right)}^2}}}$$

Standard deviation measures how spread out a distribution is, which is defined as:

(4) $${\sigma ^t} = \sqrt {{1 \over n}\sum\limits_{i = 0}^{n - 1} {{{\left( {x_i^t - {{\bar x}^t}} \right)}^2}} } $$

Correlation defines the degree to which two variables vary together, or a measure of the intensity of the association between two variables, which is defined as:

(5) $$\eqalign{ {P_{{t_1},{t_2}}} = {{Cov({t_1},{t_2})} \over {{\sigma ^{{t_1}}}{\sigma ^{{t_2}}}}} \cr \hskip-6.2pcCov({t_1},{t_2}) = E[{t_1} - E({t_1})][{t_2} - E({t_2})] \cr} $$

where ${P_{{t_1},{t_2}}}$ refers to the correlation coefficient between every two axes (*t*_1_ axis and *t*_2_ axis), *Cov*(*t*_1_, *t*_2_) refers to the covariance between every two axes, and *E*(*t*_1_) refers to a mean value of all data on the *t*_1_ axis.

#### Frequency domain features

In addition to temporal domain features, we also converted the acceleration data from its original domain (temporal domain) to the frequency domain. For any participant’s data on each axis, we calculated the mean value and the standard deviation of Power Spectral Density (PSD), which are frequency domain features. Specifically, PSD measures one signal’s power intensity in the frequency domain. The mean value of PSD represents the average power per unit of bandwidth, and the standard deviation of PSD represents the degree of dispersion in terms of power.

#### Temporal-frequency domain features

After extracting temporal and frequency domain features, we combine these two kinds of features as a third kind of features, using Fast Fourier Transform (FFT) which converts the sampled function from its original domain (time domain) to the frequency domain. The conversion is defined as:

(6) $$X_k^t = \sum\limits_{j = 0}^{n - 1} {x_j^t} {e^{ - i2\pi k{j \over n}}}\qquad k = 0, \cdots ,31$$

For any user’s data on each one axis, we run FFT analysis. In this study, we selected the first 32 amplitude coefficients as temporal-frequency domain features.

#### Feature selection

For each time series (i.e., each segment), we extract 38 features on each axis, to acquire a total of 114 features (38 × 3 = 114) (See Table 1). To improve the performance of model training, we run Principle Component Analysis (PCA) for feature selection, and we selected the features with accumulative contribution rate over 95%.

**Table 1:** All features extracted.

Features		X	Y	Z
Temporal	Skewness	1	1	1
	Kurtosis	1	1	1
	Standard deviation	1	1	1
	Correlation coefficient	1	1	1
Frequency	Mean of PSD	1	1	1
	Standard deviation of PSD	1	1	1
Temporal-frequency	FFT	32	32	32
Total		38	38	38

We calculated the Load Matrix, acquiring the factor loading between the new features and the original features. We chose the factor loading which is equal or greater than 0.71 to determine the original features mainly contributing to the new features. We found the temporal domain features (standard deviation) and temporal-frequency domain features (coefficient of FFT) are highest contribution to the new features.

### Model training

For differentiating three emotions (neutral, happy, and angry), we trained classification models using WEKA, and four different algorithms (decision tree, support vector machine, random forest, and random tree) were used to build classification models, respectively.

Decision Tree is a method commonly used in classification learning. In this study, parameters of the Decision Tree model were defined as: J48 -C 0.25 -M 2. The parameter *C* serves to set confidence threshold for pruning, and the parameter *M* serves to set minimum number of instances per leaf.

Support Vector Machine (SVM) is an algorithm, which constructs hyperplanes in a high-dimensional space for classification. In this study, parameters of the SVM model were defined as: LibSVM -S 0 -K 2. The value of parameter *S* (*S* = 0) indicates that *C* − *SVC* type of SVM is used, and the value of parameter *K* (*K* = 2) indicates that the Radial Basis Function (RBF) is selected as the kernel of SVM.

Random Forest is a meta-estimator that fits a number of decision tree classifiers on various sub-samples of the dataset. In this study, the parameter of the Random Forest model was defined as: RandomForest -I 10. The value of parameter *I* indicates that how many decision trees are constructed.

Random Tree is a method for constructing a tree or tree-map. In this approach, a tree or tree-map is formed by a stochastic process. In this study, the parameter of the Random Tree model was defined as: RandomTree -M 1.0. The parameter *M* serves to set minimum number of instances per leaf.

In this study, we applied 10-fold cross validation on training models. The performance of classification models were evaluated by examining the emotion recognition rate (*Q*), which is defined as:

(7) $$Q = {{The\ number\ of\ samples\ which\ are\ classified\ correctly} \over {The\ total\ number\ of\ samples}}$$

## Results

### Two-category classification

For two-category classification, we examined the performance of models in differentiating every two emotions. We eliminated the noise from dataset using a Moving Average Filter, and selected features using PCA. During the noise removal process, *w* is set as 3 or 5, which allows us to examine the classification performance according to *w*.

#### Differentiation between neutral and angry

For the differentiation between neutral and angry, the number of features after PCA was shown in Table 2. The classification performance is shown in Table 3.

**Table 2:** Number of features after PCA for differentiating neutral and angry.

	*w* = 3	*w* = 5
Wrist	56	49
Ankle	59	53

**Table 3:** Classification performance for differentiating neutral and angry.

		LibSVM	DecisionTree	RandomForest	RandomTree
*w* = 3	Wrist	86.0%	76.2%	71.1%	65.7%
	Ankle	72.5%	64.2%	63.8%	64.3%
*w* = 5	Wrist	91.3%	83.8%	82.8%	69.8%
	Ankle	71.3%	61.5%	62.3%	61.9%

Results showed that, for differentiating neutral from angry, the overall classification accuracy was over 61.5%. The performance of models using data collected from wrist were better than those using data collected from ankle. When we examined data collected from wrist-worn accelerometers with *w* = 5, we got the best classification accuracy (*Q* = 91.3%) using the LibSVM algorithm.

#### Differentiation between neutral and happy

For the differentiation between neutral and happy, the number of features after PCA was shown in Table 4. The classification performance can be shown in Table 5.

**Table 4:** Number of features after PCA for differentiating neutral and happy.

	*w* = 3	*w* = 5
Wrist	61	55
Ankle	61	54

**Table 5:** Classification performance for differentiating neutral and happy.

		LibSVM	DecisionTree	RandomForest	RandomTree
*w* = 3	Arist	88.5%	77.8%	64.9%	61.0%
	Ankle	80.9%	73.1%	65.7%	63.0%
*w* = 5	Wrist	78.2%	67.7%	63.1%	62.3%
	Ankle	71.7%	62.4%	61.5%	62.6%

For differentiating neutral from happy, the overall classification accuracy was over 61.0%. When we examined data collected from wrist-worn accelerometers with *w* = 3, the best classification accuracy is (*Q* = 88.5%) using LibSVM.

#### Differentiation between happy and angry

For the differentiation between happy and angry, the number of features after PCA was shown in Table 6. The classification performance can be shown in Table 7.

**Table 6:** Number of feature after PCA for differentiating neutral and happy.

	*w* = 3	*w* = 5
Wrist	56	49
Ankle	59	53

**Table 7:** The classification accuracy for differentiating happy and angry.

		LibSVM	DecisionTree	RandomForest	RandomTree
*w* = 3	wrist	88.5%	83.3%	73.8%	68.1%
	Ankle	79.1%	70.1%	65.3%	60.6%
*w* = 5	Wrist	82.5%	72.9%	66.3%	63.1%
	Ankle	71.1%	60.4%	62.0%	60.9%

For differentiating happy from angry, the overall classification accuracy was over 60.4%. When we examined data collected from wrist-worn accelerometers with *w* = 3, the best classification accuracy is (*Q* = 88.5%) on LibSVM.

### Three-category classification

Besides conducting two-category classification, we also examined the classification performance for differentiating three emotions (neutral, happy, and angry). For the three-category classification, the number of feature after PCA was shown in Table 8. The classification performance can be shown in Table 9.

**Table 8:** Number of features after PCA for differentiating neutral, happy, and angry.

	*w* = 3	*w* = 5
Wrist	56	49
Ankle	59	53

**Table 9:** Classification performance for differentiating neutral, happy, and angry.

		LibSVM	DecisionTree	RandomForest	RandomTree
*w* = 3	Wrist	79.6%	65.8%	59.0%	52.4%
	Ankle	68.6%	60.1%	53.2%	49.3%
*w* = 5	Wrist	81.2%	70.6%	66.2%	56.6%
	Ankle	62.3%	49.6%	52.4%	47.8%

Results showed that, the overall performance of three-category classification is worse than two-category classification. When we examined data collected from wrist-worn accelerometers with *w* = 5, the best classification accuracy is (*Q* = 81.2%) using LibSVM.

## Discussion

In this paper, we used wearable devices (smart bracelets) to conduct a gait analysis on 123 participants to recognize their emotions. This study demonstrates that human emotions (neutral, happy, and angry) are expressed in walking to some degree, and more importantly, a real-time recognition of human emotions (neutral, happy, and angry) can be realized by using wearable smart devices.

We found that human gait can be used to differentiate different emotions, which is consistent with previous research (Montepare, Goldstein & Clausen, 1987; Michalak et al., 2009). Results indicated that, for both two-category and three-category classification, the highest classification accuracy can reach over 88%. As humans can recognize emotions from signals (e.g. face and voice) with an accuracy of 70% − 98% (Takahashi, 2005; Bos, 2006), it suggests that classification models on gait work fairly well. In addition, SVM classifiers outperform other classifiers (e.g. Decision Tree, Random Forest, and Random Tree).

The models perform differently across different emotions. For neutral vs. non-neutral classification, it was easier to identify neutral from angry than from happy, which is consistent with other research on emotion recognition (Montepare, Goldstein & Clausen, 1987). We also found that, when the number of categories (i.e. number of emotion types) increases, the classification accuracy decreases. Probably it is partly due to an increasing complexity of the classification task. More specifically, it might be challenging to recognize various types of emotions within only one minute. If we can extend the period of observation, the classification accuracy would be increased.

The placement of wearable smart devices at different body positions may influence the performance as well. Results indicated that, in general, data collected from wrist might be much more useful to recognize human emotions.

Our method is based on cut-edge technology (Montepare, Goldstein & Clausen, 1987; Michalak et al., 2009; Sun et al., 2010). The customized smart bracelet is convenient to wear for a long time, and the procedure of acceleration data collection is non-intrusive and ecological. If we embed bluetooth in the bracelet to upload real-time walking behavioral data, we are able to monitor emotion timely, which could be used on different circumstances, such as monitoring emotion of mental disorders, children’s psychological research, etc.

It is important to note the limitations of this study. In this study, we conducted the gait analysis on a total of 123 participants, and the sample size is a bit limited. Collecting data from a larger number of participants might further validate the modeling performance. As all participants are Chinese, we do not know whether this method still works in other countries. In addition, we are not sure whether there exist cultural differences in the relationship between emotion and walking patterns. Because of the limited length of observation (1 min), we cannot compare the classification accuracy among observation periods with different lengths. Therefore, we cannot figure out the optimal observation time window for recognizing human emotions. As we took each segment as one sample, and the samples were classified independently from each other. We just looked into the classification result of each sample. In the future work, for each participant, we may use the voting method based on classification result of the segments to predict his/her emotion.

This study provides an innovative method for recognizing human emotions (neutral, happy, and angry) in an efficient manner. Through the use of wearable smart devices, we can recognize human emotions (neutral, happy, and angry) in real-time, which could be beneficial to other research including human-computer interaction.

## Conclusion

This paper built classification models for differentiating various emotions (happy, neutral, and angry), using human gait data collected from wearable smart devices. Results indicate that it is efficient to recognize human emotions (happy, neutral and angry) by using a wearable smart bracelet. This method can be helpful to realize an automatic recognition of human emotions (neutral, happy, and angry) in human-computer interaction.

## Supplemental Information

10.7717/peerj.2258/supp-1 Supplemental Information 1 Raw data.

## Additional Information and Declarations

### Data Deposition

The following information was supplied regarding data availability: The raw data has been supplied as Supplemental Dataset Files.
